# A Double-Switch Temperature-Sensitive Controlled Release Antioxidant Film Embedded with Lyophilized Nanoliposomes Encapsulating Rosemary Essential Oils for Solid Food

**DOI:** 10.3390/ma12234011

**Published:** 2019-12-03

**Authors:** Xi Chen, Qing Long, Lei Zhu, Li-Xin Lu, Li-Nan Sun, Liao Pan, Li-Jing Lu, Wei-Rong Yao

**Affiliations:** 1 Department of Packaging Engineering, Jiangnan University, Wuxi 214122, Chinasunlinan_a@163.com (L.-N.S.); breath860101@aliyun.com (L.P.);; 2 China National Center for Food Safety Risk Assessment, Beijing 100022, China; 3 Key Laboratory of Advanced Food Manufacturing Equipment and Technology of Jiangsu Province, Jiangnan University, Wuxi 214122, China; 4 State Key Laboratory of Food Science and Technology, Jiangnan University, Wuxi 214122, China; yaoweirongcn@jiangnan.edu.cn

**Keywords:** temperature-sensitive, double-switch, controlled release, antioxidant, nanoliposome, rosemary essential oils

## Abstract

In order to match the solid food oxidation during logistics and storage process under severe high temperature, a double-switch temperature-sensitive controlled release antioxidant film embedded with lyophilized nanoliposomes encapsulating rosemary essential oils (REOs) was prepared. The double switch temperature at 35.26 and 56.98 °C was achieved by development of a temperature sensitive polyurethane (TSPU) film. With biaxially oriented polyethylene terephthalate (BOPET) as a barrier layer, the intelligent complex film was prepared via coating the TSPU embedded with lyophilized nanoliposomes encapsulating REOs on BOPET. The results indicate that the REO is well encapsulated in nanoliposomes with encapsulation efficiency (*EE*) of 67.3%, high stability and lasting antioxidant effect during 60 days. The incorporation of lyophilized nanoliposomes containing REOs into TSPU remains the double-switch temperature-sensitive characteristic of the prepared TSPU. In agreement with porosity and *WVTR* results, the diffusion coefficient (*D*) of the antioxidant complex film sharply increases respectively at two switching temperatures, indicating that the intelligent double-switch temperature-sensitive controlled release property is functioning. Furthermore, compared with films directly added with REO, the lower *D*s of films added with lyophilized nanoliposomes encapsulating REOs provides a longer-lasting antioxidant activity. Thus, the acquired controlled release antioxidant film sensitive to temperature at 39.56 and 56.00 °C can be potentially applied for protection of solid food during distribution and storage process under severe high temperatures.

## 1. Introduction

Protecting food from deterioration and flavor altering caused by oxidation has become one of the major challenges for the food industry [1]. Antioxidant packaging is an effective technology to delay the oxidation process by scavenging free radical and interdicting peroxide [2]. Moreover, antioxidant film with controlled release function can provide a better antioxidant effect for the ability of adjusting the release rate of antioxidant to satisfy the demand of food to be oxidized [3]. However, most of the studies on controlled release antioxidant film focused only on the sustained release of antioxidant, in which the obtained films cannot stimulate the release rate of antioxidant according to the alteration of environmental conditions such as pH, temperature, and illumination [4]. While the oxidation rate is significantly affected by the logistics and storage environment, which may dramatically change during the distribution and storage process. Thus, it is necessary to exploit the controlled release of antioxidant films that are sensitive to environmental factors to further protect food from severe circumstances.

The environmentally responsive smart membrane can respond quickly to external environmental stimulus and convert external signals into internal signals (internal structure, pore size, etc.,) to change the performance of the materials [5]. Among all the environmental factors, temperature is one of the most significant factors influencing the oxidation process. Therefore, the academic community has demonstrated an interest in the development of temperature responsive polymer and hydrogel [6,7]. Temperature sensitive polyurethane (TSPU) is one of the excellent temperature responsive materials and has a significant phase change in a certain temperature range [8]. The phase change will lead to a sudden change of free volume, and cause the permeability change [9,10]. This character of TSPU provides a possibility to control the release speed of active substance inside the film. Hence, Dong et al. [11] applied TSPU with a single switch temperature to control the release of directly incorporated carvacrol and cinnamyl aldehyde as antimicrobials, presenting superior protection on food compared with PU without temperature sensitive property. Nevertheless, to the author’s knowledge, there has been no research on TSPU based temperature-sensitive controlled release antioxidant film. In addition, the single switch TSPU provides a relatively narrow range of stimulating temperature which may limit the application in practice. Therefore, TSPU with multiple switch temperature needs to be developed for the temperature-sensitive controlled release of antioxidants.

Among the most widely utilized antioxidant, rosemary essential oil (REO) is a typical natural antioxidant essential oil with perfect antioxidant effect and approval by FDA and European Commission [12]. Specifically, the main functional ingredients of REOs include carnosic acid, carnosol, rosmarinic acid, and rosmanol. Carnosic acid is the compound with the highest antioxidant activity in rosemary essential oil, about three times higher than carnosol and about seven times higher than synthetic antioxidants butylated hydroxytoluene (BHT) and butylated hydroxyanisole (BHA) [13]. The antioxidant activity of rosmarinic acid is stronger than that of vitamin E, and the antioxidant activity of rosmanol is about five times that of BHT and BHA. Besides, the volatile essential oils can diffuse into the space of package without contact with foods, providing a possible solution for the problem of oxidation of solid foods. Thus, REO was chosen as the antioxidant in this study. However, due to the instability and volatility of essential oils, directly blending REOs and TSPU may lead to a rapid release of antioxidant, thus nanoliposome encapsulation technique was introduced for its stable, non-toxic and sustained release properties [14]. Although the nanoliposomes encapsulating essential oils have been successfully embedded in chitosan [15,16] and protein [17] films, the incorporating of nanoliposomes containing REOs into TSPU has not been studied. Furthermore, the influence of nanoliposomes containing REOs on the structures, morphology and thermal characteristics of TSPU requires further study.

The aim of this paper was to prepare a double-switch temperature-sensitive controlled release antioxidant film based on TSPU incorporated with lyophilized nanoliposomes containing REOs as temperature sensitive layer, and BOPET as barrier layer. Then investigate how the lyophilized nanoliposomes containing REOs influence structures, morphology and thermal characteristics of TSPU, and how the double-switch TSPU control the release of REO to realize a longer-lasting antioxidant protection for solid food.

## 2. Materials and Methods

### 2.1. Materials

Polycaprolactone (PCL, *M_w_* = 4000, CP) was purchased from Suyu plastic raw materials business department (Dongguan, China). 4,4′-diphenylmethane diisocyanate (MDI, 98%) was obtained from J&K Scientific Co., Ltd. (Beijing, China). Polyethylene glycol (PEG, *M_w_* = 2000, CP), 1,4′-butanediol (BDO, AR), N,N-dimethylformamide (DMF, AR), soy lecithin (PC, BR), cholesterol (CH, ≥ 0.995), Tween 80 (T-80, CP), polyvinylpyrrolidone (PVP, ≥ 0.995) and anhydrous ethanol (GR) were bought from Sinopharm Chemical Reagent Co., Ltd. (Shanghai, China). Polyurethane (PU) was obtained from Shunte Plastic Co., Ltd. (Guangdong, China). Biaxially oriented polyethylene terephthalate (BOPET) was purchased from Yiwei Mechanical and Electrical hardware Co., Ltd. (Shanghai, China). Rosemary essential oil (REO, RG) was received from Chroma Dex Corporation (USA); 2,2-diphenyl-1-picrylhydrazyl (DPPH, ≥ 97.0%, HPLC) was acquired from Aladdin Biotechnology Co., Ltd. (Shanghai, China).

### 2.2. Preparation of Lyophilized Nanoliposomes Containing REOs

Nanoliposomes containing REOs were prepared by thin film hydration method [18]. Briefly, 1.00 g of REO, 6.00 g of soy lecithin and 1.00 g of cholesterol were fully dissolved in 150 mL of chloroform. Solvents were evaporated under reduced pressure at 30 °C using a rotary evaporator. The obtained lipid film on the inner wall was hydrated with 300 mL of phosphate buffer (pH = 6.8, 1.224 g of sodium dihydrogen phosphate and 1.584 g of disodium hydrogen phosphate) under sonication, adding 0.30 g of PVP and 9 mL of Tween 80 as surfactants. Then the obtained liposomal suspension was sonicated in an intelligent ultrasonic cell crushing machine (JYD-900; Zhixin Instrument, Co., Ltd., Shanghai, China) for 10 min. After free REOs and other impurities were removed by centrifugation at 2348.5× *g* (Centrifuge 5424; Eppendorf, Germany), suspension of nanoliposomes containing REOs was obtained by filtering through a microporous membrane. Finally, the lyophilized nanoliposomes containing REOs, with relatively uniform distribution of particle size, were obtained by freeze-drying for 36 h with pre-freezing at −80 °C for 8 h. Lyophilized nanoliposomes without REOs were prepared under the same procedure.

### 2.3. Preparation of TSPU Solution

The TSPU solution was synthesized using a two-step block copolymerization technique according to Zhou’s study [19]. Firstly, a mixture of 0.005 mol of PCL (*M_w_* = 4000) and 0.010 mol of PEG (*M_w_* = 2000) was mixed with 0.030 mol of MDI and reacted at 80 °C for 2 h to obtain TSPU pre-polymer. Additionally, DMF was added according to the viscosity of the reaction system, and the free isocyanate group (-NCO) was quantified by acetone-di-n-butylamine titration. Secondly, 0.060 mol of MDI and 0.0750 mol of BDO were added to the obtained TSPU pre-polymer for chain extension, and the reaction was carried out at 80 °C for 2 h with the ratio of -NCO and -OH fixed to 1:1, and final solid content controlled as 30% wt. Ultimately, a transparent and viscous TSPU solution was prepared.

### 2.4. Preparation of Complex Antioxidant Film

Pure TSPU film was prepared by dry phase inversion technique. The prepared TSPU solution with solid content of 55.4 g was casted on a self-made rectangular rimmed polytetrafluoroethylene (PTEF) plate of 25 cm × 15 cm, and dried at 60 °C for 24 h in a DHG-9240A electro thermostatic blast oven (Shanghai Jinghong Experimental Instrument Co., Ltd., Shanghai, China). The complex antioxidant film was prepared by coating TSPU solution incorporated with lyophilized nanoliposomes containing REOs onto BOPET through a solution casting technique, and identified as film A. The blank control film was prepared by coating TSPU solution without antioxidant onto BOPET, and identified as film B. The REO control film was prepared by coating TSPU solution directly incorporated with REOs instead of lyophilized nanoliposomes onto BOPET, and identified as film C. All the complex film (film A, B and C) were prepared with the same solid contents of TSPU (55.4 g), film area (25 cm × 15 cm) and heating condition (60 °C, 24 h) as pure TSPU film. To be comparable, the mass ratios of REOs to TSPU film in film A and film C were both controlled as 1%.

### 2.5. Quantification of REOs

Qualitative and quantitative analysis of REOs was carried out by gas chromatography-mass spectrometry (GC-MS) with a HP-5 column (GCMS-QP2010Ultra, SHIMADZU, Japan). High pure helium was used as the carrier gas with flow rate of 1.0 mL/min. The detector was acquired by electron impact with scanning range of 33~600 m/z using an ionization energy of 70 eV. 1μL of sample was injected in the split ratio of 50:1. Temperatures of injector and detector were both 280 °C. The column was initially heated at 40 °C and remained 40 °C for 3 min, then heated at 5 °C/min to 100 °C and remained at 100 °C for 8 min, subsequently heated at 5 °C/min to 190 °C and remained at 190 °C for 5 min, lastly heated at 10 °C/min to 280 °C. Since 1, 8-eucalyptol was detected to be the main component of REO, the linear regression analysis of REO quantification was related to the peak area of 1, 8-eucalyptol in this study. For REOs dissolved in anhydrous ethanol and REOs volatized in the air, the determination coefficient (*R^2^*) of the linear regression analysis was 0.9992 and 0.9910, respectively. Specially, for REOs volatized in the air, the GC-MS detection required a pretreatment of solid phase micro-extraction (SPME) at 60 °C for 0.5 h.

### 2.6. Encapsulation Efficiency and Particle Characterization

Encapsulation efficiency was determined by centrifugation techniques. In brief, the nanoliposomes in suspension and lyophilized nanoliposomes were dissolved in absolute ethanol, respectively. The encapsulated REOs were isolated by using centrifuge at 13527× *g*. Then the mass of REOs loaded in nanoliposomes, identified as *m*_1_ (mg), was measured by GC-MS and calculated by linear regression of which *R^2^* = 0.9992. Moreover, the initially mass of the loaded REOs was identified as *m*_0_ (mg). Therefore, the encapsulation efficiency (*EE*) of the nanoliposomes was calculated by Equation (1) [20].

(1) $$EE=\frac{m_{1}}{m_{0}}\times 100\%$$

The mean diameter (MD) of particle, polydispersity index (PDI) and zeta potential of nanoliposomes in suspension and lyophilized nanoliposomes were measured by Malvern Zetasizer Nano (Malvern, Worcestershire, UK). Moreover, all of the above parameters were also measured at the 10th, 20th, 40th, and 60th day to evaluate the storage stability of nanoliposomes containing REOs at 4 °C.

### 2.7. Fourier Transform Infrared (FT-IR) Spectroscopy

The FT-IR were obtained by using ALPHA-T Fourier transform infrared spectrometer (Bruker Technology Co., Ltd, Beijing, China) with transmission mode, and scanning range of 600~4000 cm^−1^. Prior to analysis, the samples were dried overnight.

### 2.8. Micro Structure Analysis

The micro structure analysis was performed by scanning electron microscopy (SEM) and atomic force microscopy (AFM). In the SEM test, for nanoliposomes, droplet samples of nanoliposome suspension were dropped onto a tin foil, after drying and gold spraying, the micro structure was observed and imaged using a SU1510 scanning electron microscope (HITACHI Ltd, Tokyo, Japan); while for films, the pretreatment was quenched in liquid nitrogen, attached to sample stub and then gold sprayed. AFM analysis was conducted through a MuLtimode8 atomic force microscope, and the pretreatment of nanoliposome suspension samples was performed by natural dry fixation.

### 2.9. Differential Scanning Calorimetry (DSC)

The DSC analysis was implemented using DSC Q2000 differential scanning calorimeter (Waters Co., Ltd, Milford, MA, USA). The test was performed under the protection of nitrogen, the temperature range was from −50 to 200 °C, and the heating rate was 10 °C/min. Each sample was tested twice, the first test was to eliminate the thermal history of TSPU sample, and the second curve was used for analysis.

### 2.10. X-ray Diffraction (XRD)

The crystallization performance analysis was obtained by X-ray diffractometer (D2 PHASER, Bruker AXS Ltd, Karlsruhe, Germany). The scanning range was 2*θ* = 5° to 40°, the scanning speed was 0.1 s/step, and the scanning step size was 0.02°.

### 2.11. Porosity Characterization

The porosity analysis of the TSPU film used the quality loss method [21]. TSPU film sample was cut to suitable size. After being soaked in anhydrous ethanol, the wet film sample was weighed and the mass was identified as *M*_2_ (g). Then the wet film sample was dried until the quality remained constant, and the weight of dried film sample was identified as *M*_1_ (g). The ratio of the pore volume to the geometric volume of the TSPU film is identified as the porosity *ε* (%), which can be calculated by Equation (2). Conduct the tests at 15, 25, 35, 45, 55, and 65 °C, respectively.

(2) $$\varepsilon =\frac{M_{2}-M_{1}}{\rho Sd}\times 100\%$$

where *ρ* is the density (g·L^−1^) of anhydrous ethanol, *S* is the area (dm^2^), and *d* is the thickness (dm) of TSPU film sample, respectively.

### 2.12. Measure of Water Vapor Transmission Rate (WVTR)

The water vapor permeability analysis of the TSPU film was conducted according to the ASTM E 96 standard [22]. Add equal amount of desiccant CaCl_2_ to 3 identical cups, respectively. Cover each cup with one piece of TSPU film and seal the cup to ensure that water vapor can only transport through the TSPU films. Then place the cups in a chamber with constant humidity of 50% RH, and constant temperature of 15, 25, 35, 45, 55, and 65 °C, respectively. Measure the mass change of CaCl_2_ in the cup after 24 h. Then, the water vapor transmission rate (*WVPR*) is calculated by Equation (3).

(3) $$WVTR=\frac{\left(W_{2}-W_{1}\right)\times 24}{t\times S}\times 100\%$$

where *W*_1_ is the initial mass of CaCl_2_ added in the cup (g), *W*_2_ is the mass of CaCl_2_ in the cup after 24 h (g), *t* is the test time (h), *S* is the area of the cup (m^2^).

### 2.13. Release Characterization

**Figure 1:**
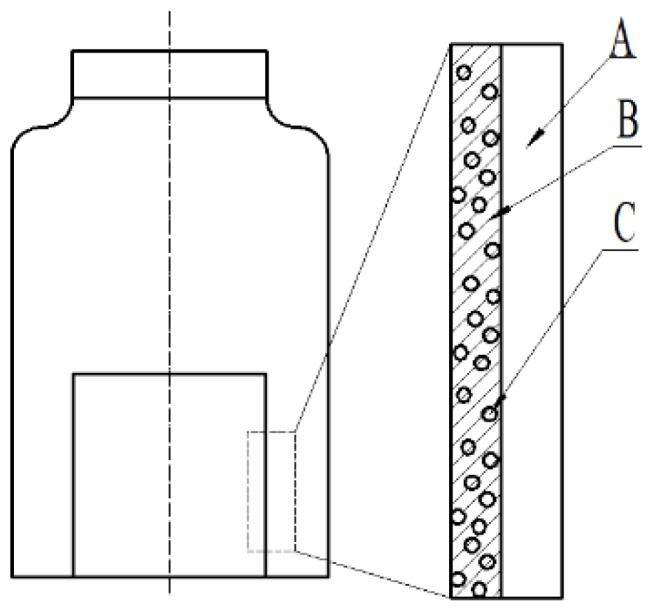
Schematic diagram of release installation. **A**: polyethylene terephthalate (PET); **B**: temperature sensitive polyurethane (TSPU); **C**: Lyophilized nanoliposomes containing rosemary essential oils (REOs).

The one-way release experiments were carried out in a release installation as shown in Figure 1 at 25, 40 and 60 °C, respectively. The one-way release was realized due to the high barrier of BOPET, thus REOs released only from the TSPU side of complex film to the air. Samples of film A and C (40 mm × 40 mm) were placed into 15 mL brown vial, respectively. Sampling occurred every 3 days with SPME method, and quantity of REO released was measured by GC-MS and calculated according to the linear regression with *R*^2^ = 0.9910.

Finally, the diffusion coefficient (*D*) of REO release was calculated by Equation (4) based on Fick’s second law with MATLAB software [23].

(4) $$\frac{M_{F,t}}{M_{F,\infty }}=1-\sum_{n=0}^{\infty }\frac{8}{{\left(2n+1\right)}^{2}\pi^{2}}\exp\left[-\frac{{\left(2n+1\right)}^{2}\pi^{2}}{4d_{p}^{2}}Dt\right]$$

where *t* is the diffusion time (s), *M_t_* is the mass of the REO (mg) in the top space of vials at time *t*, *M*_∞_ is the mass of the REO (mg) in the top space of vials at time of equilibrium, *d_p_* is the thickness (cm) of the composite film, and *D* is the diffusion coefficient (cm^2^/s) of REO.

If at the end of the experiment, the release of REO does not reach equilibrium (*M_F_*_, *t*_/*M_F_*_, ∞_ < 0.6), the diffusion coefficient *D* can be estimated by Equation (5) simplified from Equation (4). Where *M_F_*_, *p*_ is the mass of REO in the original composite film (mg). In this study, *D*s were calculated by Equation (5) for that the release of REO does not reach equilibrium (*M_F_*_, *t*_/*M_F_*_, ∞_ < 0.6).

(5) $$\frac{M_{F,t}}{M_{F,p}}=\frac{4}{d_{p}}{\left(\frac{Dt}{\pi }\right)}^{\frac{1}{2}}$$

### 2.14. Antioxidant Activity

The antioxidant activity of REO-in-nanoliposomes and REO-in-nanoliposome-in-films were determined by DPPH radical scavenging activity assay, which is suitable for the analysis of antioxidant activity of botanical drug samples. For REO-in-nanoliposomes, 2 mL of suspension of nanoliposomes containing REOs was mixed with 2 mL of 100 μmol/L DPPH ethanol solution. The mixture was mixed vigorously and then kept in a dark at room temperature for 40 min. For REO-in-nanoliposome-in-films, film A, B, and C of 40 mm × 40 mm were cut into pieces and mixed with 2 mL of 100 μmol/L DPPH ethanol solution, respectively. The mixtures were mixed vigorously and then kept in dark at room temperature for 0.5 h, 24 h, 720 h and 1440 h, respectively. Control sample was obtained though mixture of 2 mL of DPPH ethanol solution and 2 mL of pure ethanol solution. Then, the absorbance of mixture with REO and control sample were measured by UV-1800 spectrophotometer (SHIMADZU Co., Ltd, Kyoto, Japan). The DPPH radical scavenging rate can be calculated by the Equation (6) [24].

(6) $$DPPH_{S}\left(\%\right)=\frac{A_{C}-A_{S}}{A_{C}}\times 100\%$$

where *DPPH_S_* is DPPH radical scavenging rate (%), *A_s_* is the absorbance of DPPH mixed with nanoliposomes containing REOs, *A_c_* is the absorbance of DPPH in control sample.

### 2.15. Statistical Analysis

The SPSS computer program (SPSS Inc., version 22) was used to carry out the one-way analysis of variance. Differences in pairs of mean values were evaluated by the Tukey test for a confidence interval of 95%. The data is presented as means ± standard deviation.

## 3. Results and Discussion

### 3.1. Characterization of Nanoliposomes Containing REOs

**Figure 2:**
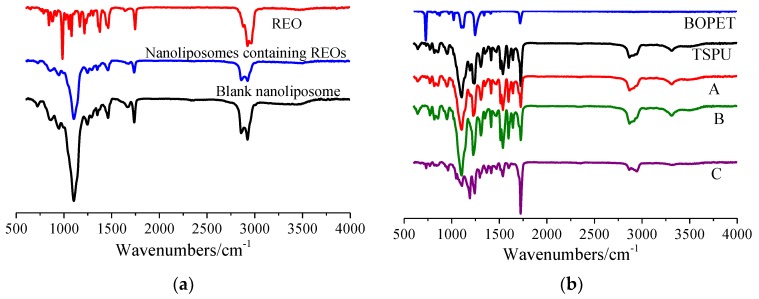
FT-IR spectrogram of REO, nanoliposomes containing REOs and blank nanoliposomes (**a**), and FT-IR spectrogram of film A, B, C, TSPU and BOPET (**b**).

FT-IR spectra of REO, nanoliposomes containing REOs and blank nanoliposomes are depicted in Figure 2a. For blank nanoliposome, the characteristic absorption peaks are mainly at 2924 and 2856 cm^−1^ (C–H, stretching vibration), 1737 cm^−1^ (C=O, stretching), 1245 cm^−1^ (symmetrical PO^2−^ stretching vibration), 1103 cm^−1^ (C–N, stretching), and 948 cm^−1^ (choline group N^+^CH_3_, stretching) [25]. The absorption bands of REO are at 2962–2881, 1745, 1464, 1411, 1376, 1215, 1080 and 984 cm^−1^, due to the stretching vibration of C–H, C = O, C = C, O–H of aromatic rings (1411 and 1376 cm^−1^), O–H of CH_2_–OH, C–O of –CH_2_–OH, and bending vibration of C–H, respectively [26]. With the addition of REO into blank nanoliposome, most absorption peaks of the REO loaded nanoliposome remain the same, implying that no chemical reaction occurs between REO and blank nanoliposome. Whilst several peaks shift slightly from 2856 to 2858 cm^−1^, 1737 to 1735 cm^-1^, and 1245 to 1247 cm^−1^, respectively, indicating that there are physical interactions between molecular groups of REO and nanoliposome.

**Figure 3:**
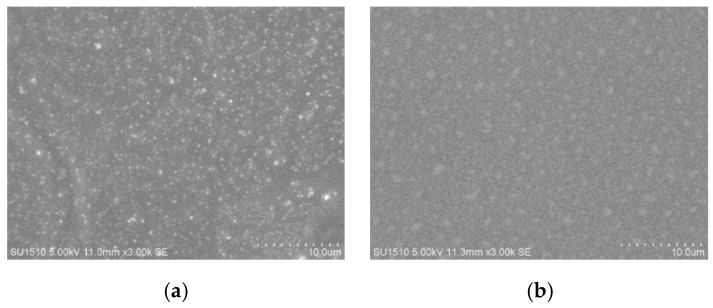

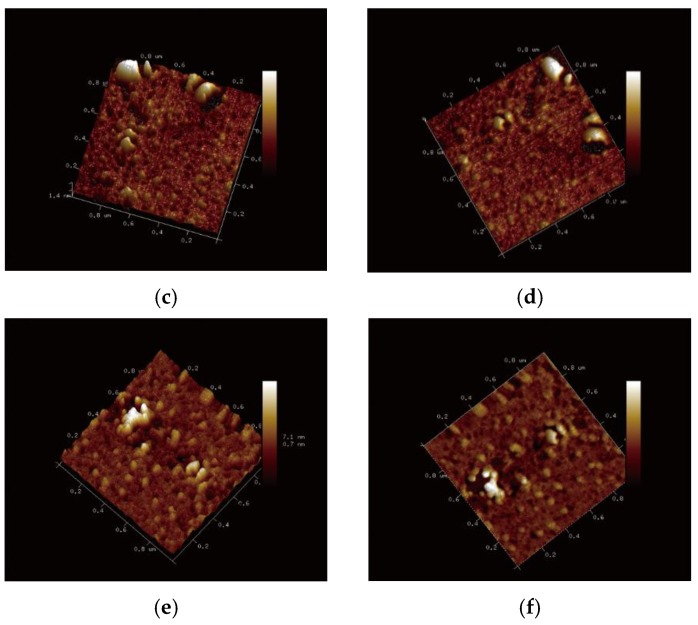
SEM images of nanoliposomes containing REOs (**a**) and blank nanoliposomes (**b**), and AFM images of nanoliposomes containing REOs (**c**,**d**) and blank nanoliposomes (**e**,**f**).

SEM and AFM images of nanoliposomes containing REOs and blank nanoliposomes are presented in Figure 3a. It can be observed from Figure 3a that all the nanoliposomes are regular and smooth spherical vesicles with uniform size and homogeneous dispersion, without obvious aggregation phenomenon.

The *EE*, MD, PDI, Zeta potential and antioxidant activity results of nanoliposomes containing REOs are shown in Table 1. The encapsulation efficiency is an important index for evaluating the effect of encapsulating. With a good repeatability, the *EE* of nanoliposomes freshly prepared is 67.34%, and the decrease of *EE* is 0.82% after 40 days of storage. The results present an effective encapsulation and extraordinary storage stability. Normally, MD in the range of 50 and 200 nm [27], PDI between 0 and 0.3 [28], and absolute value of Zeta potential greater than 30 mV, means a more stable and uniform dispersion of nanoliposome. Moreover, the smaller the PDI means more concentrated particle size and more stable system. In this research, MD, PDI, and Zeta potential results of nanoliposomes freshly prepared and stored respectively over 10, 20, 40 and 60 days are in the range of 60.75~64.57, 0.230~0.236, and −31.83~−30.02, respectively. All the results are in the intervals that represent good stability and uniform dispersion of nanoliposome system. The radical scavenging rate of DPPH with absorbance around 0.356 ranges from 61.22% to 55.77% with a slight decline tendency as time consuming, indicating relatively high radical scavenging activity. Besides, the relatively minus change of the above indexes indicates excellent storage stability over 60 days. 

### 3.2. Double Switch Temperature Sensitive Characterization

**Figure 4:**
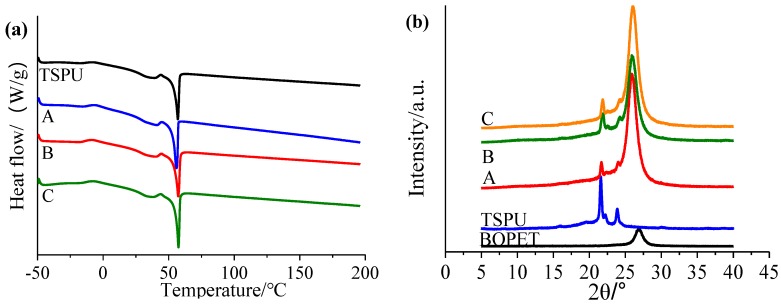
DSC curves of film A, B, C, TSPU (**a**), and XRD patterns of film A, B, C, TSPU, BOPET (**b**).

DSC curves of film A, B, C, TSPU are presented in Figure 4a, where TSPU DSC curve shows two phase transition temperature (crystalline and melting peaks) of 35.26 °C and 56.98 °C, caused by temperature sensitive property of PEG2000 and PCL4000 soft segment, respectively, indicating a smart membrane with double temperature switch is acquired. There are also two similar phase transition temperature (crystalline melting peaks) of film A (39.56 °C, 56.00 °C), film B (36.04 °C, 57.32 °C), and film C (35.26 °C, 57.49 °C), respectively. The results demonstrate that the coating of TSPU onto BOPET (film B), the addition of REO (film C) and the incorporation of nanoliposomes containing REOs (film A) remain the double-switch temperature-sensitive characteristic of TSPU film obtained in this study. In addition, compared with the acquired TSPU, the lower phase transition temperature of film A increases slightly from 35.26 to 39.56 °C. This could be attributed to the enhanced rigidity and limited motion of PEG2000 soft segment resulting from the interaction between nanoliposomes and PEG2000 soft segment.

XRD patterns of film A, B, C, TSPU and BOPET are depicted in Figure 4b. XRD pattern of TSPU has peaks of 2*θ* mainly at 21.6°, 22.2 and 23.9° which correspond to the crystalline peaks of the soft segment and the hard segment [29], indicating that the hard segment MDI does not destroy the crystalline morphology of the soft segment PEG and PCL. There are also two similar dominant characteristic peaks of 2*θ* of film A (21.7°, 24.0°), film B (21.9°, 24.2°), and film C (21.9°, 24.1°), respectively. The results imply that coating of TSPU onto BOPET (film B), the addition of REO (film C) and the incorporation of nanoliposomes containing REOs (film A) does not disrupt the intrinsic crystalline morphology of TSPU film obtained in this study. This is in accordance with DSC results that the TSPU based complex films still have temperature response characteristics. Furthermore, compared with pure TSPU film, the peak intensity of film A, B and C all decrease apparently, demonstrating a decrease of crystallinity of the soft segment or hard segment. Besides, all the BOPET based films have similar peak of 2*θ* at 26.0° (film A), 26.0° (film B) and 26.1° (film C) compared with BOPET (26.9°), implying that film A, B and C remain the crystalline morphology of pure BOPET film. Moreover, with increase of peak intensity, the crystallinity of film A, B and C increase compared with pure BOPET film.

**Figure 5:**
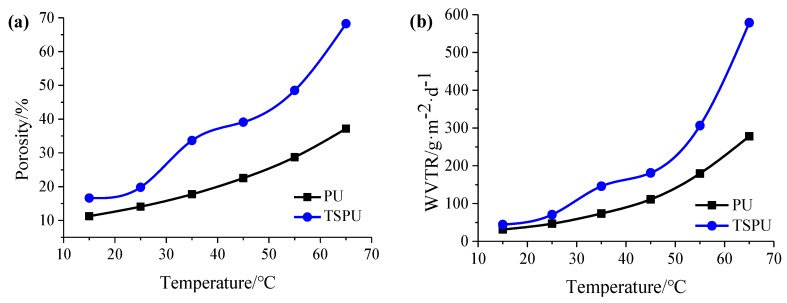
Porosity of TSPU and PU films (**a**), and *WVTR*s of TSPU and PU films (**b**).

Porosity *ε* and *WVTR* of TSPU and PU films at different temperatures ranging from 15 °C to 65 °C are described in Figure 5a,b, respectively. The diagram shows that both porosity and *WVTR* of the control film PU without temperature sensibility rise slightly as regular with the increase of temperature. However, the porosity and *WVTR* of TSPU film undergo two irregular significant increase around phase transition temperature of 35.26 °C and 56.98 °C. Specifically, the porosity of TSPU increase from 19.81% at 25 °C to 33.67% at 35 °C and from 48.49% at 55 °C to 68.24% at 65 °C, showing two significant improvements as high as 170% and 141%, respectively. Consistent with the porosity increase tendency, the *WVTR* of TSPU increase from 70.49 g/m^2^·24 h at 25 °C to 145.71 g/m^2^·24 h at 35 °C and from 306.32 g/m^2^·24 h at 55 °C to 578.54 g/m^2^·24 h at 65 °C, showing two significant improvements as high as 207% and 189%, respectively. The results indicate that with the phase transition of PEG2000 and PCL4000 soft segment in TSPU controlled by temperature, the porosity representing the size of the free volume hole in TSPU and micro-Brownian motion in the polymer [30] alters rigorously, thus the *WVTR* of TSPU changes in the same tendency. In this case, *WVTR* of TSPU determined by porosity illustrates that the TSPU film fabricated in this study has an intelligent temperature response characteristic with a double switch.

### 3.3. Characterization of Controlled Release Complex Film

Figure 2b is the FT-IR spectra of film A, B, C, TSPU, and BOPET. For TSPU, absorption peak at 3311 cm^−1^ assigns to the stretching vibration of hydroxyl group O–H and asymmetric and symmetric stretching of N–H, peak at 2946 and 2867 cm^−1^ assigns to the bending and stretching vibration of C–H, peaks at 1537, 1305, 1237, 1105, 1019 and 952 cm^−1^ correspond to the bending vibration of N–H, stretching vibration of O–H of –CH_2_–OH (1305 and 1237 cm^−1^), stretching of C–N, C–O, and bending vibration of =C–H, respectively. There is no characteristic absorption peak of –NCO in the range of 2260-2280 cm^−1^, while an absorption peak appears at 1725 cm^−1^, indicating that carbamate group (-NHCOO-) has been produced through the nucleophilic reaction between –NCO and –OH [8]. The incorporation of REO loaded lyophilized nanoliposomes into TSPU does not alter most of the characteristic peak of TSPU except minus shifts of 3311 to 3313 cm^−1^, 1305 to 1307 cm^−1^, 1237 to 1227 cm^−1^, and 952 to 950 cm^−1^, respectively. This reveals that no chemical bond is newly built up between REO loaded lyophilized nanoliposomes and TSPU, while between which exists a weak physical interaction. In the contrast, the direct incorporation of REO into TSPU dramatically changes some of the characteristic peaks of TSPU. The peak at 3311 cm^−1^ not only shifts to 3323 cm^−1^, but also substantially decreases the intensity of peak. The peaks at 1305, 1019 and 952 cm^−1^ also have a relatively large scaled shift to 1294, 1046 and 960 cm^−1^. The results indicate that the physical interaction between REO and TSPU is stronger than which between lyophilized nanoliposomes encapsulating REO and TSPU.

**Figure 6:**
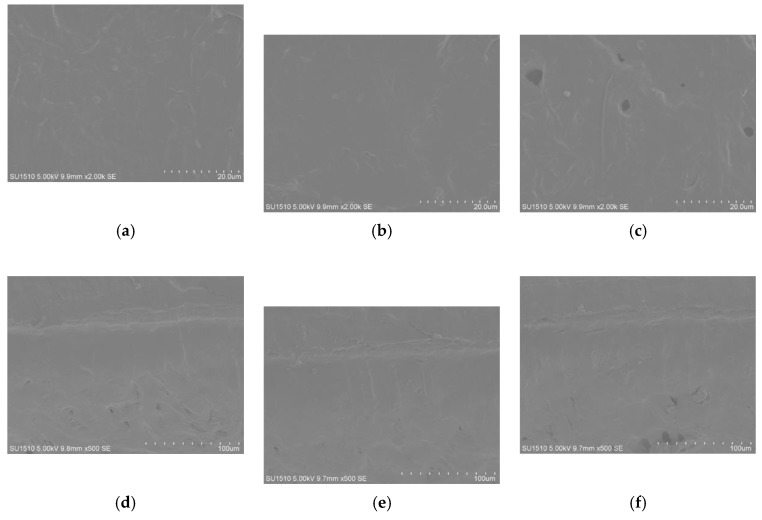
SEM surface pictures of TSPU side of film A, B and C (**a**–**c**), and cross-section of film A, B and C (**d**–**f**), respectively.

SEM pictures performing surface micromorphology of TSPU side and cross-section of film A, B and C are exhibited in Figure 6. It can be observed from the TSPU side surface images that film B as blank control film is relatively dense and smooth, while film A incorporated with nanoliposomes containing REOs is slightly uneven with aggregated nanoliposomes and small amount of micropores, and film C as REO control film has more micropores compared with film A. The micropores might be formed by the volatilization of REO during the film drying process. Moreover, the less micropores of film A compared with film C is probably due to the decline of REO volatilization, resulting from the reinforced interaction between the nanoliposomes and the soft segment in TSPU film in agreement with DSC results in this study (Section 3.2). Likewise, the amount of micropores presented in the cross-section image of film A, B and C obey the same rule as in the TSPU side surface image, while the distribution of micropores is more in the center and less on the surface. In addition, the cross-section morphology of all the three films present a good consistency without delamination at the interface of TSPU and BOPET, indicating that the TSPU layer is well bonded to the BOPET layer.

**Figure 7:**
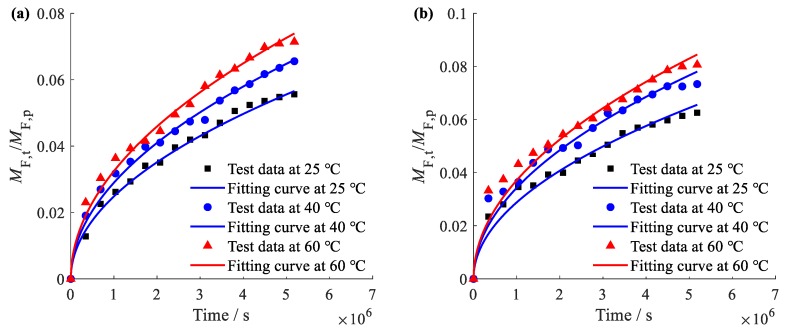

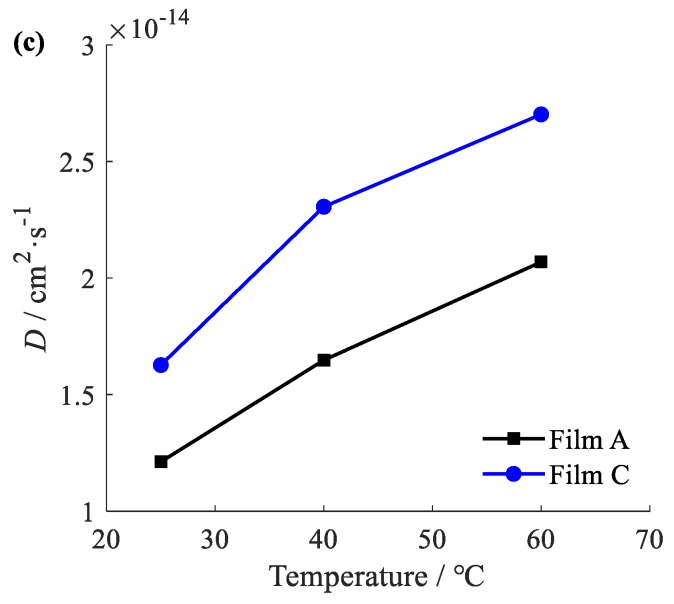
Test data and fitting curves of REO release from film C (**a**) and film A (**b**) at 25, 40 and 60 °C, respectively, and *D* of film C and A at 25, 40 and 60 °C, respectively (**c**).

Both the test data and fitting curves of REO releasing proportion out of film C and A at 25 °C, 40 °C and 60 °C are depicted in Figure 7a,b, respectively. The goodness of fit is evaluated by means of the root mean square error (RMSE) listed in Table 2. The results show that the model satisfactorily fits the test data, suggesting the adopted model is suitable for characterization of REO release from the complex film. The calculated *D*s are presented in Table 2, and the increase tendency of *D* with temperature ascending is described in Figure 7c.

It can be observed that the release of REO from both films (film C and A) at all temperatures (25 °C, 40 °C and 60 °C) has not reached equilibrium in 60 days, and the mass rate of released REO was less than 10%, indicating a very slow release of REO. This is in accordance with the low value of *D* at magnitude of 10^−14^ (cm^2^·s^−1^). Moreover, *D*s of film A are smaller than that of film C at each temperature tested, implying a significant barrier and protection effect of nanoliposome layer in this study. Although *D*s of film C are larger than *D*s of film A at each temperature tested, the relatively low release rate of REO represented by *D* at magnitude of 10^−14^ (cm^2^·s^−1^) is probably caused by the strong interaction between directly added REO and TSPU as discussed before according to FT-IR results. Furthermore, with the temperature ascending, *D* of both film C and A undergoes an abnormal dramatically increase (Figure 7c) against Arrhenius theory, in which *D* is exponentially related to temperature. This demonstrates that both complex film C and A have remained the double switch temperature sensitive property of TSPU, and boost the release around phase transition temperature 39.56 °C and 56.00 °C. The intelligent temperature-sensitive controlled release function is realized via enlarging the free volume hole size and accelerating micro-Brownian motion of soft segment in TSPU.

The results of the DPPH radical scavenging test for film A, B and C are shown in Table 3. It can be concluded from Table 3 that the DPPH radical scavenging activity of film A gradually increases during the period of 1440 h (60 d). The extremely low *DPPHs* of film B might be caused by experimental error. For film C, the DPPH free radical scavenging activity is higher within 720 h (30 d) whereas lower at 1440 h (60 d) compared with film A. The results demonstrate that film A has a lasting antioxidant effect due to the controlled release property of nanoliposomes. Besides, the obtained multicomponent film can be recycled by solving isolation method according to research of Patrizia et al. [31].

## 4. Conclusions

In this study, a TSPU film with double switch temperature at 35.26 °C and 56.98 °C was prepared by copolymerization of PEG2000 and PCL4000 soft segment. With BOPET as a barrier layer, an intelligent double-switch temperature-sensitive controlled release antioxidant film was fabricated via coating the prepared TSPU embedded with lyophilized nanoliposomes encapsulating REOs on BOPET. The results indicate that the REO is well encapsulated in nanoliposomes with *EE* of 67.34%, high stability and lasting antioxidant effect during 60 days. The incorporation of nanoliposomes containing REOs into TSPU does not destroy the double-switch temperature-sensitive characteristic of the prepared TSPU. In agreement with porosity and *WVTR* results, the diffusion coefficient *D* of the antioxidant complex film sharply increases respectively at two switching temperatures, indicating that the intelligent double switch temperature-sensitive controlled release property is functioning. Furthermore, compared with films directly added with REO, the lower Ds of films added with lyophilized nanoliposomes encapsulating REOs provides a longer-lasting antioxidant activity. In general, the acquired controlled release antioxidant film sensitive to temperature at 39.56 °C and 56.00 °C can be potentially applied for the protection of solid food during the distribution and storage process under severe high temperatures.

## Figures and Tables

**Table 1:** Encapsulating effect, antioxidant activity and storage stability of nanoliposomes containing REOs at 4°C. Means followed by the same letter in a column are not significantly different from each other at *P* < 0.05.

Time/d	MD/nm	(s. d.)	PDI	(s. d.)	Zeta Potential/mV	(s. d.)	*EE*/%	(s. d.)	*DPPHs*/%	(s. d.)
0	60.75 ^a^	1.079	0.230 ^a^	0.0074	−31.83 ^a^	0.7767	67.34 ^a^	1.649	61.22 ^a^	1.427
10	61.15 ^a^	2.734	0.231 ^a^	0.0099	−31.76 ^a^	0.6737	67.21 ^a^	4.695	60.57 ^a^	1.484
20	61.23 ^a^	2.314	0.231 ^a^	0.0069	−31.32 ^a^	0.7263	66.70 ^ab^	2.574	59.80 ^a^	0.5814
40	62.39 ^a^	2.658	0.233 ^a^	0.0083	−30.56 ^a^	0.1227	66.52 ^ab^	0.4148	58.93 ^a^	0.2998
60	64.57 ^a^	1.22	0.236 ^a^	0.0018	−30.02 ^a^	0.3998	64.92 ^b^	0.4344	55.77 ^b^	0.3976

**Table 2:** Diffusion coefficient *D* of REO release from film A and C at different temperatures. Means followed by the same letter in a column are not significantly different from each other at *P* < 0.05.

Film Samples	*D*/×10^−14^ (cm^2^·s^−1^)					
	25 °C	RMSE	40 °C	RMSE	60 °C	RMSE
A	1.212 ^a^	0.001220	1.648 ^a^	0.001509	2.069 ^a^	0.001967
C	1.627 ^a^	0.002744	2.306 ^a^	0.003596	2.702 ^a^	0.004054

**Table 3:** DPPH radical scavenging activity of film A, B and C. Means followed by the same letter in a column are not significantly different from each other at *P* < 0.05.

Film Samples	Absorbance								DPPHs/%							
	0.5 h	(s. d.)	24 h	(s. d.)	720 h	(s. d.)	1440 h	(s. d.)	0.5 h	(s. d.)	24 h	(s. d.)	720 h	(s. d.)	1440 h	(s. d.)
Control	0.918 ^a^	0.0223	0.918 ^a^	0.0026	0.917 ^a^	0.0066	0.917 ^a^	0.0194	0.000 ^a^	0.0000	0.000 ^a^	0.0000	0.000 ^a^	0.0000	0.000 ^a^	0.0000
A	0.585 ^b^	0.0142	0.525 ^b^	0.0017	0.481 ^b^	0.0067	0.435 ^b^	0.0085	36.27 ^b^	0.8533	42.81 ^b^	0.2892	47.55 ^b^	1.663	52.56 ^b^	0.1818
B	0.904 ^a^	0.0019	0.906 ^a^	0.009	0.902 ^a^	0.0246	0.901 ^a^	0.0637	1.530 ^a^	0.0055	1.300 ^c^	0.0015	1.640 ^a^	0.0055	1.740 ^c^	0.0122
C	0.512 ^c^	0.0178	0.463 ^c^	0.0328	0.472 ^b^	0.0028	0.526 ^b^	0.0251	44.23 ^c^	1.019	49.57 ^d^	0.6615	48.53 ^b^	1.01	42.64 ^d^	0.2782
